# Factors Affecting Nurses' Performance of Noise Management in Adult Intensive Care Units

**DOI:** 10.1155/2023/9886888

**Published:** 2023-12-29

**Authors:** Seo Jeong Kim, Haeyoung Min

**Affiliations:** ^1^Surgical Intensive Care Unit, Gyeongsang National University Hospital, 79 Gangnam-ro, Jinju, Gyeongnam 52727, Republic of Korea; ^2^College of Nursing, Gyeongsang National University, 816-15 Jinju-daero, Jinju, Gyeongnam 52727, Republic of Korea

## Abstract

The aim of this study was to identify factors affecting the noise management performance of adult intensive care unit (ICU) nurses in Korea. The ICU environment is exposed to various types of noises due to the use of medical devices for the treatment of critically ill patients. The noise has an adverse effect on nurses' health and their nursing performance, which might potentially threaten patient safety. A cross-sectional study was conducted with a total of 148 nurses working in adult ICUs from two university hospitals in Korea. Data were collected using self-administered questionnaires, which included various aspects such as noise knowledge, response to noise, patient safety culture, and noise management performance. Multiple regression analysis revealed that teamwork (*β* = 0.33, *p*=0.004), patient safety policy, and procedure (*β* = 0.25, *p*=0.037), which were subscales of patient safety culture, as well as frequency of noise experience (*β* = 0.16, *p*=0.030) were significant factors affecting adult ICU nurses' performance of noise management. This study provides a significant basis for promoting adult ICU nurses' performance in noise management. A structured policy and procedure for noise management should be established for reducing noise in adult ICUs. In terms of nursing education, it is necessary to consider and develop team-based training programs to promote noise management performance in the ICU. Nurse managers and nursing organizations should consider how to create conditions in which nurses can recognize the importance of noise reduction and perform well in noise management.

## 1. Introduction

Noise in the intensive care unit (ICU) is a result of various sources, including staff activities and the use of medical devices, along with structural noise from the air conditioning, heating, and ventilation systems [1]. The ICU environment is exposed to various types of noises due to the constant use of medical devices for 24-hour intensive monitoring and treatment of critically ill patients, as well as the presence of healthcare providers [2]. Noise is an unwanted sound from the subjective point of view of an individual [3] and adversely affects the physiological and psychological state of an individual or group [4]. According to the noise management guidelines of the World Health Organization (WHO), the average noise level in an ICU should not exceed 35 dBA during the day, 30 dBA at night, and a maximum of 40 dBA overall, as hospital noise may interfere with the recovery of patients [5]. However, in Korea, the average noise level at an adult ICU was 55 dBA or higher [6].

The most representative causes of noise in the ICU were found to be staff conversations and medical device alarms [6–8]. Nurses who are constantly exposed to high levels of noise in the ICU may experience the risks of hearing loss, anxiety, stress, burnout, fatigue, decreased concentration and performance, decreased attention span, and impaired judgment [9]. In addition, the effects of noise in the ICU may impede communication between healthcare providers and patients and delay the awareness and response of nurses to medical device alarms, threatening patient safety [10]. Thus, effective noise management is important for both nurses and patients.

Noise management performance (NMP) defines the extent to which efforts have been made to reduce or control noise within the past month [11]. One factor known to influence poor noise management in ICUs is the lack of nurses' knowledge regarding noise in the ICU and its effects on the health of staff and patients [12]. Knowledge about noise in the ICU refers to the level of knowledge regarding all noise-related matters, such as the WHO hospital noise guidelines, effects of noise in the ICU, noise level in the ICU, and the main noise sources in the ICU [13]. It is, therefore, essential for nurses managing the ICU environment to be aware of the noise present there, which can facilitate effective noise reduction [14].

Patient safety culture is defined as the beliefs, values, and behavior patterns shared at the organizational, departmental, and individual levels to minimize the harm to patients that may occur in the process of providing healthcare services [15]. Excessive noise in the ICU may lead to stress, fatigue, distraction, and inefficient communication for nurses and doctors, creating an unsafe environment that is prone to medical errors and threatening patient safety [16]. Nurses' perceptions of patient safety culture influence their attitudes and behaviors related to patient safety performance [17].

Despite the importance of noise management, only few studies have been conducted on this topic in Korea. Previous studies have focused on noise levels in the ICU and patients' perceptions of noise [18], noise level by type in adult ICUs [6], the development of noise reduction interventions [13], and noise experience and response to noise among patients and nursing staff in the ICU [19]. In Western countries, many studies related to ICU noise have been conducted on the sources of noise and effectiveness of noise reduction strategies [20], the effect of noise on ICU patients' sleep quality [21] and nurses' health [4], effect of noise reduction on ICU patients' delirium [22], and so on. However, little is known about the factors affecting the NMP of adult ICU nurses in both Korea and Western countries. Past studies showed that a higher level of patient safety culture in ICU nurses was associated with higher patient safety performance [23, 24] and better alarm management performance [25, 26], as well as a lower incidence of patient safety accidents [27].

Therefore, this study aims to (1) investigate the level of experience of noise, knowledge of noise, response to noise, patient safety culture, and noise management performance of nurses working in adult ICUs in Korea and (2) identify the factors affecting the NMP of adult ICU nurses in Korea.

## 2. Materials and Methods

### 2.1. Study Design and Sample

A cross-sectional survey design was used in this study. A convenience sampling method was used to recruit nurses working in five adult ICUs from two university-affiliated general hospitals in Korea. One of the hospitals included in the study had a total of 899 beds, with 46 adult ICU beds (including medical, surgical, emergency, and cardiovascular ICUs). The second hospital had a total of 577 beds and 30 adult ICU beds dedicated to integrated ICUs. The nurse-to-patient ratio in the adult ICUs of both hospitals was in the range of 1 : 2∼3. The nurses provided care to patients with respiratory, kidney, and cardiovascular issues, as well as those recovering from surgery or procedures. In both hospitals, the distance between beds in the adult ICUs was 1.5 m and the distance between the wall and the bed was 1 m. Neither hospital had implemented noise reduction facilities or devices.

The inclusion criteria were being registered nurses who had worked full time for more than three months in adult ICUs. Nurses who did not provide direct care to patients (e.g., nurse managers) were excluded.

The sample size required for this study was calculated using the G^*∗*^ power 3.1.9.4 program. The minimum sample size was 141 based on an effect size of 0.15, *α* of 0.05, a power of 0.90, and nine independent variables in a multiple regression. The target sample size was 155, considering a dropout rate of 10%.

### 2.2. Data Collection and Recruitment

Data were collected from April 1 to April 20, 2022. After the study was approved, the chief of nursing and department head nurses in both hospitals were provided with information about the study's background and purpose in order to obtain their cooperation and approval for data collection. Participants were recruited by using information sheets in the adult ICUs at the two hospitals. Nurses were informed about the purpose of the study, potential risks and benefits of participation, confidentiality, and the right to withdraw from the study at any time. Once the nurses agreed to participate, they signed the consent form. The principal investigator (PI) then distributed printed questionnaires to the participants, which included demographic information and five different tools. The questionnaires were in Korean. Each participant was provided a sealed opaque double envelope containing a research description explaining the purpose of the study, the confidentiality of responses, and the assurance that any personally identifiable information would not be disclosed and used only for research purposes. The envelope also contained the questionnaire. After completing the questionnaire, participants returned it directly to the researcher in a sealed state to maintain anonymity. Nurses were provided the option to complete the questionnaire either before or after their working hours, and it took approximately 20 to 30 minutes for them to fill out the questionnaire. Participants received a small gift as a token of appreciation. A total of 152 questionnaires were obtained, with four being excluded due to insufficient responses. Consequently, the final data analysis included 98 nurses from one hospital and 50 nurses from the other hospital, comprising a total of 148 nurses who had worked across three shifts.

### 2.3. Data Collection Tools

#### 2.3.1. Noise Experience

Noise experience was measured using the noise experience scale developed in 2021 by Yun et al. [19]. The content validity of this scale was supported by an expert panel review; the content validity index of the scale (scale-CVI) was 0.98 [19]. The tool assesses the frequency of experience and level of noise for each noise source, consisting of a total of 45 items: 35 items for human factors, seven items for medical device factors, and three items for environmental factors. The frequency of experience for each noise source was scored on a four-point Likert scale (1 = “not at all” to 4 = “always”). Higher scores indicate higher frequencies of experience from the noise source. Cronbach's *α* was 0.97 for all items in Yun et al.'s [19] study and 0.98 in this study. The noise level for each noise source was scored at 10 points (0 = “no” to 10 = “extremely high”). Higher scores indicate higher levels of noise perceived. Cronbach's *α* was 0.97 in Yun et al.'s study [19].

#### 2.3.2. Noise Knowledge

Noise knowledge was measured using the noise knowledge scale developed in 2020 by Yun, Kwak, and Yoo [13]. The content validity of this scale was supported by an expert panel review; the scale-CVI was 0.94 [13]. The tool consists of a total of 54 items. Each correct answer was scored as 1 point, and each wrong or “I don't know” answer was scored as 0. Higher scores indicate higher levels of knowledge about noise. The Kuder–Richardson Formula 20 (KR-20) value was 0.91 in Yun et al.'s [13] study.

#### 2.3.3. Response to Noise

Response to noise was measured using the response to noise scale developed in 1994 by Son [28] and modified in 2020 by Yun et al. [13]. The content validity of the original scale was supported by an expert panel review; however, the scale-CVI was not reported [28]. Moreover, the content validity of the modified tool was supported by an expert panel review; the scale-CVI was 0.92 [13]. The tool consists of a total of 30 items: 17 items for physiological response and 13 items for emotional response, for a total of 10 points (0 = “strongly disagree” to 10 = “strongly agree”). Higher scores indicate higher levels of response to noise. Cronbach's *α* was 0.95 in Yun et al.'s [13] study.

#### 2.3.4. Patient Safety Culture

Patient safety culture was measured using the patient safety culture scale developed in 2015 by Lee [15]. The content validity of this scale was supported by an expert panel review; the scale-CVI was 0.96. The construct validity was supported by confirmatory factor analysis, explaining 56.3% of the total variance for seven factors [15]. The tool consists of a total of 35 items in seven subscales with nine items for leadership, six items for teamwork, five items for patient safety knowledge/attitude, four items for patient safety policy/procedure, four items for a nonpunitive environment, four items for a patient safety improvement system, and three items for patient safety priority. Each item was scored on a five-point Likert scale (1 = “strongly disagree” to 5 = “strongly agree”). Higher scores indicate higher levels of patient safety culture. Cronbach's *α* was 0.93 in Lee's [15] study.

#### 2.3.5. Noise Management Performance

Noise management performance was measured using the NMP scale developed in 2019 by Kim, Son, and Kang [11]. The original scale was developed based on the neonatal ICU environment, consisting of a total of 18 items. The content validity of this scale was supported by an expert panel review; however, the scale-CVI was not reported [11]. We modified the scale based on literature related to ICU noise. The modified scale was validated through an expert panel review, consisting of one nursing professor, five ICU head nurses, and two ICU nurses with more than 10 years of nursing experience. Each item was scored on a 4-point Likert scale (1 = “not relevant,” 2 = “somewhat relevant,” 3 = “quite relevant,” and 4 = “highly relevant”), and the CVI was calculated. Each item's CVI was calculated by experts, giving a rating of either 3 or 4 on the 4-point scale. The scale-CVI was calculated using the average of the item-CVI scores. The item-CVI ranged from 0.87 to 0.90 and the scale-CVI was 0.96, indicating good validity [29]. The final modified scale included a total of 23 items and each item was scored on a 5-point Likert scale (1 = “never” to 5 = “always”). Higher scores indicate higher levels of NMP. Cronbach's *α* was 0.73 in Kim et al.'s [11] study.

### 2.4. Data Analysis

The data from this study were analyzed using IBM SPSS Statistics for Windows, Version 27.0. Armonk, NY: IBM Corp [30]. Descriptive statistics were used to assess the characteristics of participants and variables. An independent *t*-test and one-way ANOVA were used to analyze the differences in NMP, according to participants' characteristics followed by a post-hoc test using Scheffé's test. Pearson's correlation coefficient was used to examine the correlations between NMP and variables. Finally, a hierarchical multiple regression analysis was conducted to examine the factors affecting the NMP of adult ICU nurses.

### 2.5. Ethical Considerations

This study was approved by the Institutional Review Board of each hospital (No. 2021-08-026-003 and No. 2021-09-009-004). Informed consent was obtained from all study participants.

## 3. Results

### 3.1. Characteristics of Study Participants

A total of 148 nurses participated in this study. The mean age of the participants was 28.36 ± 4.29 years. Most participants were female (91.9%), unmarried (77.0%), and had a bachelor's degree or lower (90.5%). The participant's mean years of nursing experience were 5.52 ± 4.34 years, and their mean years of adult ICU experience were 4.23 ± 2.30 years. Only 35 of the 148 participants (23.6%) reported the implementation of quiet time in the ICU, which refers to the practice of reducing therapeutic activities that contribute to noise, dimming the lights, and speaking softly during the night shift compared to the day and evening shifts in order to encourage inpatients to sleep well. The majority of nurses who implemented quiet time (65.7%) did so between 12 a.m. and 5 a.m., while approximately half of them (51.4%) implemented quiet time between 12 a.m. and 6 a.m. Regarding noise management education, most participants (99.3%) had not received any education for ICU noise management. Most participants (75.7%) reported that noise management education was needed (see Table 1).

### 3.2. Descriptive Statistics of Variables

Table 2 shows the mean scores of noise experience, noise knowledge, response to noise, patient safety culture, and NMP. The mean score of the frequency of experience with noise sources was 3.06 ± 0.62, and the highest mean score was for medical device factors (3.41 ± 0.62). The mean score of the noise level for noise sources was 4.74 ± 1.55, and the highest mean score was for medical device factors (5.96 ± 1.87). The mean score of noise knowledge was 28.91 ± 9.98, with a correct answer rate of 54%. The mean score of response to noise was 4.89 ± 2.32 and that of emotional response to noise (5.42 ± 2.55) was greater than that of physiological response (4.22 ± 2.29). The mean score of patient safety culture was 3.52 ± 0.49; the highest mean score was for patient safety knowledge/attitude was 3.86 ± 0.66, and the lowest mean score was for patient safety priority (2.86 ± 0.70). The mean score of the NMP was 3.45 ± 0.64. Additionally, the mean scores of the frequency of experience and noise level for each noise source are included in Supplementary 1.

### 3.3. Reliability of Tools

In this study, the reliability of the tools was evaluated using Cronbach's *α* and KR-20 values. The sample size was appropriate to assess the tools' reliability based on the recommendation of Kennedy [31]. Cronbach's *α* was 0.97 for noise experience, 0.98 for response to noise, 0.93 for patient safety culture, and 0.91 for NMP. The KR-20 value was 0.93 for noise knowledge, indicating high reliability and internal consistency of the measurement tools used in the study.

### 3.4. Differences in NMP According to Participants' Characteristics

As shown in Table 3, there was a significant difference between NMP and the need for noise management education (*t* = 2.66, *p*=0.009). No significant difference was found in other participants' characteristics.

### 3.5. Correlation between NMP and Factors

The NMP was correlated with the frequency of noise experience (*r* = 0.20, *p*=0.013), noise knowledge (*r* = 0.21, *p*=0.009), response to noise (*r* = 0.23, *p*=0.005), and patient safety culture (*r* = 0.50, *p* < 0.001) (see Table 4).

### 3.6. Factors Affecting NMP

Factors affecting the NMP of adult ICU nurses were identified using multiple regression analysis. The need for noise management education was input into Model 1. Variables such as the frequency of noise experience, response to noise, noise knowledge, and patient safety culture were input into Model 2. The result of the Durbin–Watson statistic was 2.330, suggesting no autocorrelation between error terms. As a result of calculating the standardized residuals, all values were found to be within ±3 except for two values, assuming a normal distribution of error terms. The tolerance was 0.32 to 0.88 and the variance expansion factor (VIF) was 1.13 to 3.16, indicating no multicollinearity between the independent variables.

As a result of the analysis, the variable that had a significant effect on NMP in Model 1 was the need for noise management education (*β* = 0.22, *p*=0.007), explaining 4.0% of the variance. In Model 2, the variables that significantly affected NMP included teamwork (*β* = 0.33, *p*=0.004) and patient safety policy and procedure (*β* = 0.25, *p*=0.037), which were subscales of patient safety culture, as well as frequency of noise experience (*β* = 0.16 *p*=0.030), explaining 33.9% of the variance (see Table 5).

## 4. Discussion

This study aimed to investigate the level of experience with noise, knowledge of noise, response to noise, patient safety culture, and NMP among nurses working in adult ICUs in Korea and to identify the factors affecting the NMP of these nurses. Among the major findings of the study, the noise generated by medical devices was identified as a major noise source experienced by ICU nurses. Moreover, nurses tended to respond more emotionally than physically to the noise. Finally, factors such as teamwork, patient safety policies and procedures related to patient safety culture, and the frequency of noise experience were found to be significant factors affecting the NMP of adult ICU nurses in Korea.

This study revealed the noise sources of adult ICUs, nurses' responses to noise, and factors affecting the NMP of adult ICU nurses. The major source of noise in an adult ICU, which was experienced and perceived by nurses, was associated with medical device factors such as alarm sounds from various machines. These results were consistent with those of previous studies [18, 19]. Kim and Park [18] reported that the highest average noise was at 73.2 dBA for the operating sounds of the nebulizer, followed by syringe pump alarms at 72.6 dBA, and the cause of noise at night being the sounds from medical devices. Bach, Berglund, and Turk [32] found that 80 to 99% of medical device alarms were incorrectly activated. The easiest and most practical way to reduce the occurrence of false alarms in medical devices is to individualize alarm settings for the condition of each patient [2]. Therefore, interventions are needed to manage and improve the medical device alarm system in adult ICUs in association with human, technical, and organizational factors.

Consistent with previous research [19], nurses' emotional response to noise was greater than their physiological response to it. Exposure to noise in the ICU may cause psychological reactions such as anxiety, stress, misjudgment, decreased work performance and concentration, and communication disturbance in ICU nurses [10], as well as physiological responses such as tension, headaches, fatigue [33], and increased heart rate [34]. Since noise in the ICU negatively affects not only the behavior of nurses but also overall health, including physiological, emotional, and cognitive health [35], it is necessary to implement robust approaches to reduce noise in the ICU.

The nurses' mean score of NMP was above average, suggesting that ICU nurses were aware of the importance of noise management and tried to reduce noise for patients. Our results are hard to compare with previous studies due to the scarcity of studies using the same measure. However, Yun et al. [13] found that the mean score of NMP increased after the noise reduction intervention program for ICU nurses. Thus, effective strategies and efforts to reduce noise in adult ICUs are needed.

In this study, there was a significant difference in nurses' NMP according to the need for noise management education, which was higher in the group that agreed with this need than in the group that disagreed. However, it was difficult to compare these results due to the absence of previous studies on the same topic. Nevertheless, considering that all participants, except one, did not have any experience in noise management education in this study, most participants seemed to have agreed with the need for education.

This study found that teamwork, patient safety policy and procedure, and the frequency of experience with noise sources were significant factors affecting the NMP of adult ICU nurses. The most influential factor was nurses' teamwork, which was a domain of patient safety culture. Teamwork involves cooperation that supports and encourages one another to adhere to patient safety principles in conducting work and participate directly in improving patient safety [15]. Johansson et al. [12] suggested that discussing noise issues through regular team meetings and encouraging and sharing noise reduction actions were a good way to reduce noise in the ICU. A high level of teamwork has a positive effect on achieving treatment goals by coordinating nursing work performance, managing the treatment process, and connecting patients and nurses [36]. Therefore, achieving noise reduction as a common goal for the entire ICU team can be an effective strategy.

Patient safety policy and procedure, a domain of patient safety culture, were identified as the second influencing factor in NMP. Patient safety policy and procedure are structured to effectively prevent medical errors and are well communicated and established at the clinic [15]. To ensure patient safety, it is necessary to develop a system that minimizes the harm that errors cause to patients [37]. Graham and Cvach [38] reported that standardization of monitor alarm training at the hospital level and implementation of the monitoring protocol increased the monitor alarm management performance of ICU nurses and reduced false alarms by 43% compared to before the interventions. In addition, the Joint Commission [39] suggested the establishment of a safe alarm sound system according to the situation of medical institutions, recommending the establishment of systematic policies and procedures for alarm sound management. Thus, it is necessary to establish patient safety policy and procedure related to noise at the hospital level to create an organizational patient safety culture and to manage noise reduction effectively.

The frequency of experience with noise sources was found to be the third influencing factor for NMP. In this study, the frequency of experience with noise sources was the highest for medical device factors. Lewandowska et al. [40] reported that healthcare providers exposed to frequent medical device alarms deviated from the original purpose of the alarms, experienced disruption in their workflow, and made frequent mistakes due to omission of confirmation, negligence, and poor concentration. However, this is inconsistent with the results of this study. In this study, the highest score of NMP was for “I made an effort to immediately resolve the medical device alarms,” followed by “If the medical device made an inappropriate sound, I tried to fix it immediately or requested a repair,” and “When various bells were ringing, I tried to resolve them immediately.” Moreover, in this study, the major source of noise included medical device factors, which also had a high level of NMP. This finding indicates that a higher frequency of experience with each noise source among adult ICU nurses is associated with a higher level of NMP for the noise source. However, further study is needed to determine the effect of the frequency of experience with noise sources on NMP in the adult ICU.

### 4.1. Implications for Nursing Management

The study findings suggest that teamwork and patient safety policy and procedure-related patient safety culture, as well as the frequency of experience with noise sources, are significant determinants of adult ICU nurses' performance in noise management. This supports the need of collaborative work with hospital staffs to reduce noise. Nurse managers and nursing organizations can discuss noise issues with other hospital staffs to inform the importance of noise reduction for patients. Furthermore, we recommend the development of team-based training programs to promote NMP in the ICU.

Organizational support is needed to build the policy and procedure for noise management. Establishing structured policies and procedures for noise management can help facilitate effective communication between healthcare providers and other hospital staffs. In addition, a standardized guideline in noise management, including noise assessment, intervention, and prevention, is necessary to improve ICU nurses' performance in noise management.

In this study, increasing the frequency of nurses' experience with noise sources was associated with increasing nurses' performance in noise management. However, previous research reported that frequent exposures to medical device alarms could cause alarm fatigue and decreased sensitivity to alarms that could lead to inappropriate responses to meaningful alarms. It is unclear whether nurses who are exposed to frequent noise perform better with noise management. More research is required to examine the correlation between nurses' performance in noise management and the frequency of noise experience.

This study is significant in that it investigated the level of experience in noise, knowledge of noise, response to noise, patient safety culture, and NMP of nurses working in the adult ICU. It also identified the factors impacting nurses' performance in noise management. The findings of this study provide significant evidence that highlights the importance of teamwork, patient safety culture, and patient safety policies and procedures in improving the NMP of adult ICU nurses. These findings provide a basis for the development of a noise reduction intervention program based on a team approach. In addition, at the hospital level, patient safety policies and procedures related to hospital noise need to be established, and noise management guidelines suitable for adult ICUs need to be developed and applied in practice.

### 4.2. Limitations

Although the present study reveals important findings, it has several limitations. First, because this study was cross-sectional in design, it might not provide strong evidence for causality between NMP, teamwork, patient safety culture, and patient safety policies and procedures. Second, this study used a convenience sampling method to recruit participants from two university hospitals in one province of Korea. Therefore, the study participants might not be representative of all Korean adult ICU nurses or those from other countries, which limits the generalizability of the findings. Further research involving adult ICU nurses recruited from hospitals of various regions, sizes, and countries should be conducted. Third, since data were collected using self-reporting tools, it may be subjected to recall bias and other inaccuracies. Further research observing nurses' NMP might be needed to determine how well nurses perform in noise management. Finally, although the modified tool used in this study was validated by an expert panel review using a CVI, further research is needed to evaluate the tool's overall validity.

## 5. Conclusions

The findings of this study indicate that the primary source of noise experienced by nurses in the adult ICU is related to medical devices, particularly the alarm sounds originating from various machines. Moreover, nurses who were exposed to such noise reported both physical and emotional reactions, which can potentially affect their work performance. These findings highlight the importance of effective noise management in ICUs, implying that ICU nurses should recognize the significance of minimizing noise levels for the wellbeing of patients. In addition, this study found that the main factors affecting adult ICU nurses' performance of noise management were teamwork, patient safety policy and procedure, and the frequency of experience with noise sources. This study provides a significant basis for establishing the policy and procedure of noise management in preventing noise for adult ICU patients. Moreover, these results will allow researchers to develop effective training programs that promote teamwork and the performance of noise management for adult ICU nurses. We believe that this study holds meaningful implications for future research and the development of practical interventions aimed at noise reduction in the adult ICU.

## Figures and Tables

**Table 1:** Characteristics of study participants (*N* = 148).

Characteristic	Category	*n* (%)	M ± SD
Age (years)	<25	30 (20.3)	28.36 ± 4.29
	25 to 29	75 (50.7)	
	30 to 34	22 (14.9)	
	≥35	21 (14.2)	

Gender	Female	136 (91.9)	
	Male	12 (8.1)	

Marital status	Unmarried	114 (77.0)	
	Married	34 (23.0)	

Education level	Bachelor's or lower	134 (90.5)	
	Master's or higher	14 (9.5)	

Position	Staff nurse	143 (96.6)	
	Charge nurse	5 (3.4)	

Working experiences as a RN (years)	<1	15 (10.1)	5.52 ± 4.34
	1 to <3	43 (29.1)	
	3 to <5	21 (14.2)	
	≥5	69 (46.4)	

Adult ICU experience (years)	<1	20 (13.5)	4.23 ± 2.30
	1 to <3	47 (31.8)	
	3 to <5	29 (19.6)	
	≥5	52 (35.1)	

Working unit	Medical ICU	41 (27.7)	
	Surgical ICU	36 (24.3)	
	Emergency ICU	30 (20.3)	
	Integrated ICU	29 (19.6)	
	Cardiovascular ICU	12 (8.1)	

Quiet time	Yes	35 (23.6)	
	No	113 (76.4)	

Experience of noise management education	Yes	1 (0.7)	
	No	147 (99.3)	

Need for noise management education	Yes	112 (75.7)	
	No	36 (24.3)	

*Note.* RN = registered nurse.

**Table 2:** Descriptive statistics of variables (*N* = 148).

Variable		Range	M ± SD
*Noise experience*			
Frequency of experience for noise source	Human factor	1–4	3.00 ± 0.64
	Medical device factor		3.41 ± 0.62
	Environmental factor		0.97 ± 0.83
	Total		3.06 ± 0.62
Noise level for noise source	Human factor	0–10	4.55 ± 1.54
	Medical device factor		5.96 ± 1.87
	Environmental factor		4.73 ± 1.55
	Total		4.74 ± 1.55

Noise knowledge		0-1	0.54 ± 0.18
Response to noise	Physiological	0–10	4.22 ± 2.29
	Emotional		5.42 ± 2.55
	Total		4.89 ± 2.32
Patient safety	Leadership	1–5	3.62 ± 0.71
	Teamwork		3.72 ± 0.63
	Patient safety knowledge/attitude		3.86 ± 0.66
	Patient safety policy/procedure		3.53 ± 0.72
	Nonpunitive environment		3.26 ± 0.83
	Patient safety improvement system		3.30 ± 0.74
	Patient safety priority		2.86 ± 0.70
	Total		3.52 ± 0.49

Noise management performance		1–5	3.45 ± 0.64

**Table 3:** Difference in noise management performance according to participants' characteristics (*N* = 148).

Characteristic	Category	M ± SD	*t*/*F*	*p*
Age (years)	<25	3.56 ± 0.51	0.77	0.514
	25 to 29	3.41 ± 0.62		
	30 to 34	3.35 ± 0.80		
	≥35	3.56 ± 0.71		

Gender	Female	3.48 ± 0.64	1.60	0.111
	Male	3.17 ± 0.66		

Marital status	Unmarried	3.48 ± 0.65	0.33	0.744
	Married	3.44 ± 0.64		

Education level	Bachelor's or lower	3.45 ± 0.64	−0.15	0.880
	Master's or higher	3.48 ± 0.65		

Position	Staff nurse	3.44 ± 0.65	1.02	0.308
	Charge nurse	3.74 ± 0.32		

Working experiences as a RN (years)	<1	3.45 ± 0.57	1.33	0.267
	1 to <3	3.59 ± 0.49		
	3 to <5	3.27 ± 0.67		
	≥5	3.42 ± 0.72		

Adult ICU experience (years)	<1	3.42 ± 0.82	0.59	0.622
	1 to <3	3.42 ± 0.82		
	3 to <5	3.34 ± 0.55		
	≥5	3.44 ± 0.68		

Working unit	Medical ICU	3.44 ± 0.60	0.09	0.986
	Surgical ICU	3.41 ± 0.66		
	Emergency ICU	3.49 ± 0.51		
	Integrated ICU	3.47 ± 0.68		
	Cardiovascular ICU	3.49 ± 0.98		

Quiet time	Yes	3.54 ± 0.57	0.92	0.358
	No	3.42 ± 0.66		

Need for noise management education	Yes	3.53 ± 0.59	2.66	0.009
	No	3.21 ± 0.74		

**Table 4:** Correlation between noise management performance and independent variables (*N* = 148).

	1	2	3	4	5	6	7	8	9	10	11	12	13	14	15
(1) Frequency of experience for noise source	1														

(2) Noise level for noise source	0.475 (<0.001)	1													

*Response to noise*															
(3) Physiological	0.265 (0.001)	0.537 (<0.001)	1												
(4) Emotional	0.357 (<0.001)	0.491 (<0.001)	0.809 (<0.001)	1											
(5) Total	0.334 (<0.001)	0.536 (<0.001)	0.933 (<0.001)	0.966 (<0.001)	1										

(6) Noise knowledge	0.204 (0.013)	0.258 (0.002)	0.455 (<0.001)	0.415 (<0.001)	0.454 (<0.001)	1									

*Patient safety*															
(7) Leadership	0.050 (0.546)	0.005 (0.955)	0.069 (0.405)	0.048 (0.563)	0.060 (0.472)	0.091 (0.270)	1								
(8) Teamwork	−0.024 (0.773)	−0.060 (0.469)	0.109 (0.189)	0.004 (0.962)	0.050 (0.545)	0.166 (0.044)	0.743 (<0.001)	1							
(9) Patient safety knowledge/attitude	0.142 (0.085)	0.047 (0.568)	0.103 (0.215)	0.117 (0.156)	0.117 (0.158)	0.194 (0.018)	0.607 (<0.001)	0.657 (<0.001)	1						
(10) Patient safety policy/procedure	0.015 (0.856)	0.079 (0.337)	0.145 (0.078)	0.022 (0.790)	0.077 (0.351)	0.102 (0.216)	0.704 (<0.001)	0.690 (<0.001)	0.670 (<0.001)	1					
(11) Nonpunitive environment	−0.059 (0.479)	−0.080 (0.334)	−0.113 (0.173)	−0.066 (0.429)	−0.089 (0.279)	0.126 (0.128)	−0.016 (0.843)	0.044 (0.593)	0.136 (0.100)	−0.038 (0.645)	1				
(12) Patient safety improvement system	0.037 (0.656)	0.106 (0.201)	0.115 (0.163)	−0.014 (0.864)	0.042 (0.613)	0.109 (0.188)	0.597 (<0.001)	0.578 (<0.001)	0.498 (<0.001)	0.697 (<0.001)	−0.083 (0.315)	1			
(13) Patient safety priority	−0.051 (0.540)	−0.185 (0.025)	−0.154 (0.062)	−0.196 (0.017)	−0.187 (0.023)	0.044 (0.598)	−0.100 (0.229)	0.057 (0.494)	−0.032 (0.703)	−0.076 (0.359)	0.475 (<0.001)	−0.137 (0.096)	1		
(14) Total	0.032 (0.697)	−0.009 (0.917)	0.074 (0.373)	0.000 (0.92)	0.036 (0.663)	0.175 (0.034)	0.886 (<0.001)	0.861 (<0.001)	0.789 (<0.001)	0.821 (<0.001)	0.261 (0.001)	0.708 (<0.001)	0.147 (0.075)	1	

(15) Noise management performance	0.203 (0.013)	0.098 (0.237)	0.290 (<0.001)	0.170 (0.039)	0.231 (0.005)	0.213 (0.009)	0.391 (<0.001)	0.502 (<0.001)	0.452 (<0.001)	0.503 (<0.001)	−0.050 (0.542)	0.423 (<0.001)	0.086 (0.297)	0.505 (<0.001)	1

**Table 5:** Hierarchical multiple linear regression analysis (*N* = 148).

Variable	Model 1					Model 2				
	*B*	SE	*β*	*t*	*p*	*B*	SE	*β*	*t*	*p*
(Constant)	3.21	0.10		30.57	<0.001	0.63	0.35		1.80	0.074
Need for noise management education (reference = no)	0.32	0.12	0.21	2.66	0.009	0.12	0.11	0.08	1.14	0.255
Frequency of noise experience						0.17	0.08	0.16	2.19	0.030
Response to noise						0.03	0.02	0.11	1.38	0.169
Noise knowledge						0.09	0.27	0.03	0.33	0.746
*Patient safety culture*										
Leadership						−0.013	0.10	−0.14	−1.26	0.210
Teamwork						0.34	0.12	0.33	2.91	0.004
Patient safety knowledge/attitude						0.05	0.10	0.05	0.46	0.643
Patient safety policy/procedure						0.22	0.11	0.25	2.10	0.037
Patient safety improvement system						0.08	0.08	0.09	0.95	0.346
Adj-*R*^2^			0.040					0.339		
*R* ^2^			0.046					0.379		
*F*(*p*)			7.06 (0.009)					9.36 (<0.001)		

## Data Availability

The data supporting the study findings are available from the corresponding author upon reasonable request. The data are not publicly available due to privacy or ethical restrictions.

## Supplementary Materials

Supplementary Materials See Table S1 in the Supplementary Materials for comprehensive analysis.
